# Preventive Treatment with Ketamine Attenuates the Ischaemia-Reperfusion Response in a Chronic Postischaemia Pain Model

**DOI:** 10.1155/2015/380403

**Published:** 2015-06-16

**Authors:** Suryamin Liman, Chi Wai Cheung, Kar Lok Wong, Wai Tai, Qiu Qiu, Kwok Fu Ng, Siu Wai Choi, Michael Irwin

**Affiliations:** ^1^Department of Anaesthesiology, The University of Hong Kong, Pokfulam, Hong Kong; ^2^Laboratory and Clinical Research Institute for Pain, The University of Hong Kong, Pokfulam, Hong Kong; ^3^Department of Anaesthesiology, China Medical University and Hospital, Taichung 40402, Taiwan

## Abstract

Ischemia and inflammation may be pathophysiological mechanisms of complex regional pain syndrome (CRPS). Ketamine has proposed anti-inflammatory effects and has been used for treating CRPS. This study aimed to evaluate anti-inflammatory and analgesic effects of ketamine after ischaemia-reperfusion injury in a chronic postischaemia pain (CPIP) model of CRPS-I. Using this model, ischemia was induced in the hindlimbs of male Sprague-Dawley rats. Ketamine, methylprednisolone, or saline was administered immediately after reperfusion. Physical effects, (oedema, temperature, and mechanical and cold allodynia) in the bilateral hindpaws, were assessed from 48 hours after reperfusion. Fewer (56%) rats in the ketamine group developed CPIP at the 48th hour after reperfusion (nonsignificant). Ketamine treated rats showed a significantly lower temperature in the ischaemic hindpaw compared to saline (*P* < 0.01) and methylprednisolone (*P* < 0.05) groups. Mechanical and cold allodynia were significantly lower in the ischaemic side in the ketamine group (*P* < 0.05). Proinflammatory cytokines TNF-*α* and IL-2 were significantly lower at the 48th hour after reperfusion in ketamine and methylprednisolone groups, compared to saline (all *P* < 0.05). In conclusion, immediate administration of ketamine after an ischaemia-reperfusion injury can alleviate pain and inflammation in the CPIP model and has potential to treat postischaemic pain.

## 1. Introduction

Complex regional pain syndrome (CRPS) is a chronic neuropathic pain disorder. Its key features in humans include spontaneous pain, hyperalgesia, allodynia, and abnormal vasomotor and sudomotor activities [1]. The incidence is about 26.2 per 100,000 persons and it is more prevalent among females than males [2]. CRPS is divided into two types: CRPS-II involves nerve injury while CRPS-I does not. The pathophysiological mechanisms of CRPS remain poorly understood but the clinical features suggest that the pathogenesis involves inflammation, ischaemia, nerve regeneration, and abnormal cross talk between affected nerves and blood vessels that are characterized by complicated cellular and molecular changes [3–5]. The blister fluid of CRPS patients has high levels of interleukin- (IL-) 6 and tumour necrosis factor-alpha (TNF-*α*) [6]. Interleukin- (IL-) 2 and TNF-*α* are also elevated systemically in patients with CRPS-I [7]. Thus, drugs modulating the cytokine system have started to be used for CRPS pain management in clinical trials [8].

Ketamine, an N-methyl-D-aspartate (NMDA) receptor antagonist and dissociative anaesthetic, has been shown to produce analgesia by inhibiting both normal and pathologic pain pathways [9]. Activated by glutamate, the NMDA receptor is believed to play an important role in the development of central sensitization, which can induce chronic pain, including CRPS. NMDA receptor antagonism may attenuate central sensitization and further reduce the symptoms of CRPS. In addition, ketamine has an anti-inflammatory effect [10], which may partially contribute to its analgesic properties. It has been used to treat CRPS clinically and shows promise in this area [11]. However, to date, there has been no study conducted to evaluate its anti-inflammatory analgesic effects in pain conditions. In this study, we also compared the anti-inflammatory effects of ketamine with methylprednisolone (a glucocorticoid anti-inflammatory agent), which served as a positive control, to investigate whether ketamine had additional therapeutic effects compared to methylprednisolone on a postischaemic pain model.

We hypothesized that if administered early after ischaemia-reperfusion injury, ketamine would modify the postischaemic responses, including pain and inflammation, in the CPIP animal model established by Coderre and colleagues [12]. The model has been shown to mimic CRPS-I reliably by 3-hour inductions of ischaemia and reperfusion [12], presenting hyperaemia, plasma extravasation, mechanical and cold allodynia, and the induction of TNF-*α*, IL-2, IL-6, and nuclear factor kappa B (NF*κ*B) [13].

## 2. Materials and Methods

All procedures were carried out according to the US National Institutes of Health Guide for the Care and Use of Laboratory Animals and were approved by the Committee on the Use of Live Animals in Teaching and Research at the University of Hong Kong. The license to conduct experiments was issued by the Department of Health, the Government of the Hong Kong Special Administrative Region.

### 2.1. Animal Preparation

There were three treatment groups in this study: a ketamine treatment group (group KE), a methylprednisolone (corticosteroid) treatment group (group MP), and a 0.9% saline treatment group (group NS). Ten 270~300 g adult male SD rats (Charles River Laboratories, USA) were used for each group based on our preliminary study (unpublished). The animals were housed individually in isolated cages with food and water available ad libitum, on a 12 : 12 h light : dark cycle in the laboratory animal unit at the University of Hong Kong. The room temperature was maintained at 23°C and humidity ranged between 25% and 45%.

### 2.2. CPIP Criteria

Successful development of CPIP relies on the existence of mechanical allodynia, which meets the criteria of a 30% decrease in the mechanical threshold of an ischaemic limb (ipsilateral side) at the forty-eighth hour after reperfusion [12]. Those fulfilling the criteria would be regarded as having successfully developed CPIP. The proportion of rats meeting the criteria for successful development of CPIP was calculated. Since effects on the modification of the postischaemic responses (anti-inflammatory and analgesic effects) of early ketamine administration were assessed, all of the rats in this study were recruited to evaluate physical signs, pain behaviour, and serum proinflammatory cytokine levels.

### 2.3. CPIP Model and Drug Administration

The rats were initially anaesthetised with intraperitoneal (i.p.) pentobarbital 40 mg/kg, followed by 13 mg per hour for the first hour and 6.5 mg for the second hour. Three doses were given in total. To induce ischaemia, a tourniquet (Nitrile 70 Durometer O-ring) with 7/32-inch internal diameter was placed around the rat's left hindlimb (ipsilateral) near the ankle joint and proximal to the medial malleolus. The O-rings were selected to produce a tight-fit that produced ischaemia similar to that produced by a blood pressure of 350 mmHg and were left on the limb for 3 hours. Blood flow to the limb was confirmed with laser Doppler [12]. Briefly, a laser Doppler probe (DP1T-V2; Moor Instruments, Axminster, UK) and a fibre were loosely taped to the plantar surface of the ipsilateral paw. Blood flow was recorded with a DRT4 monitor (Moor Instruments, Axminster, UK). At the third hour, the O-ring was cut and reperfusion occurred. The termination of sodium pentobarbital anaesthesia was timed so that the rats recovered fully within 30–60 minutes following reperfusion. The study drugs ketamine (100 mg/kg), methylprednisolone (30 mg/kg), and 0.9% saline in a bolus of 0.5 mL were given intraperitoneally, immediately after the removal of the O-ring tourniquet. Dose of ketamine was chosen according to a previous study on intestinal ischaemia-reperfusion injury [14]. Methylprednisolone was used as a positive control and the dose was determined according to a study of methylprednisolone on ischaemia-reperfusion injury in rat livers [15].

### 2.4. Physical Signs and Behaviour Assessment

The rats were taken to the laboratory platform for one hour per day for a total of two days prior to the experiment and 30 minutes before behaviour assessment on the experiment day to become accustomed to the laboratory environment. The investigator assessing the physical signs and pain behaviour of the rats was blinded to the study medications administered.

### 2.5. Hindpaw Temperature

Coderre's CPIP animal model showed an increase in skin temperature in the ischaemic hindpaw lasting for two hours after reperfusion [12]. A thermocouple wire was used to measure the baseline temperature of both hindpaws before ischaemia and from the fifth minute to the seventh day after the reperfusion. Three sites were tested over the dorsum of the hindpaw. These were the spaces between the first and second metatarsals (medial), the second and the third metatarsals (central), and the fourth and fifth metatarsals (lateral) in medial, central, and lateral sequence. The three measurements were averaged to obtain a mean temperature for each side.

### 2.6. Hindpaw Thickness

The thickness of the hindpaw was measured as an indication of hindpaw volume and oedema after induction of CPIP, as it has been reported that the hindpaw volume can be presented as hindpaw thickness [16]. In this experiment, a manual calliper was used to measure the ventral thickness of both hindpaws (maximum dorsal) at baseline before ischaemia and from the fifth minute to the seventh day after reperfusion. The calliper was lightly applied to the skin without tissue displacement.

### 2.7. Mechanical Allodynia

The criterion for successful development of CPIP is a 30% reduction in the mechanical allodynia threshold on the ipsilateral hindpaw. This was measured by assessing the hindpaw withdrawal threshold with a von Frey fibre of an Electrovonfrey apparatus (IITC/Life Science Instruments). A blunt fibre with a stiffness of 65 g was applied against the hindpaw plantar skin at approximately the midsole, avoiding the tori pads. It was pushed until it is slightly bent, and a hindpaw withdrawal within 6–8 seconds of the stimulus was considered a positive response. The force (in grams) that was needed to elicit a positive response was then recorded and shown on the screen of the Electrovonfrey apparatus. The withdrawal threshold was measured at 5- to 10-minute intervals, alternating between the right and left sides until each side had been tested twice. The readings of the ischaemic and contralateral sides were averaged to give a mean withdrawal threshold for each side. This was measured at baseline before ischaemia and from the sixth hour to the seventh day after reperfusion.

### 2.8. Cold Allodynia

Cold allodynia was measured as a decrease in withdrawal latency to acetone, according to Coderre et al. [12]. The measurements started at baseline before ischaemia and from the sixth hour to the seventh day after reperfusion. The rats were placed in a clear plastic cylinder on a glass surface maintained at a constant temperature of 23°C. After 15 minutes of acclimatization, a drop of acetone was placed on the skin of the heel. Unlike normal rats which usually ignore the stimulus, CPIP rats often respond to it with an exaggerated withdrawal that we were able to time. The hindpaw withdrawal latency was repeatedly measured at 5–10-minute intervals, alternating between the right and left sides until each side had been tested twice. The two withdrawal latencies of both ischemic and contralateral sides were averaged to give mean withdrawal latency.

### 2.9. Heat Threshold

Heat threshold was assessed by timing hindpaw withdrawal latency to radiant heat using a fabricated radiant heat device according to Vatine et al. [17]. The time measurements started at baseline before ischemia and from the sixth hour to the seventh day after reperfusion. Rats were placed in a clear plastic cylinder on a glass surface maintained at a constant temperature of 23°C. After 15 minutes of acclimatization, a radiant light source was focused on the heel of the ischaemic side hindpaw. The hindpaw withdrawal latency was measured at 5–10-minute intervals, alternating between the right and left sides, until each side had been tested twice. The two withdrawal latencies of both ischaemic and contralateral sides were averaged to give mean withdrawal latency.

### 2.10. Serum Proinflammatory Cytokines

An increase in proinflammatory cytokines can indicate the involvement of the inflammatory process in CPIP. Blood was collected from the tail vein forty-eight hours after reperfusion. After being stored overnight at 4°C, the blood was centrifuged at 1000 ×g for 20 minutes. The supernatant was taken and allocated for use. Serum TNF-*α* and IL-2 were measured by an enzyme-linked immunosorbent assay (ELISA) and commercial assays (R&D Systems, Inc., Minneapolis).

### 2.11. Statistical Analysis

A two-way repeated measures analysis of variance (ANOVA) was used to compare temperature, hindpaw thickness, withdrawal threshold to von Frey fibre and acetone, and heat thresholds in a manner that ensured the data were compared on a complete study time-course basis instead of individual time points. One-way ANOVA was used to test the serum inflammatory cytokines. When a significant result was obtained, Tukey's test was applied for post hoc comparisons. The proportions of mortality and CPIP development among groups were tested by Fisher's exact test. Data are presented as mean ± S.E.M, and differences are considered significant at a *P* value ≤0.05.

## 3. Results

### 3.1. Mortality Rate and Proportion of Rats with Successful Development of CPIP

One animal in the ketamine and two in the methylprednisolone treatment groups died after the injection of the study drug, making the residual number of each group *n* = 9 in group KE, *n* = 8 in group MP, and *n* = 10 in group NS with no significant difference in the mortality rate among the three groups.

Using the criteria of a 30% decrease in the withdrawal threshold of the von Frey fibre (mechanical allodynia) on the ischaemic side, the percentages of successful CPIP development were 56% in the KE group, 75% in the MP group, and 80% in the NS group. Although KE group had the least development rate of CPIP, no statistically significant difference among the three treatment groups could be found.

### 3.2. Hindpaw Temperature and Thickness

**Figure 1:**
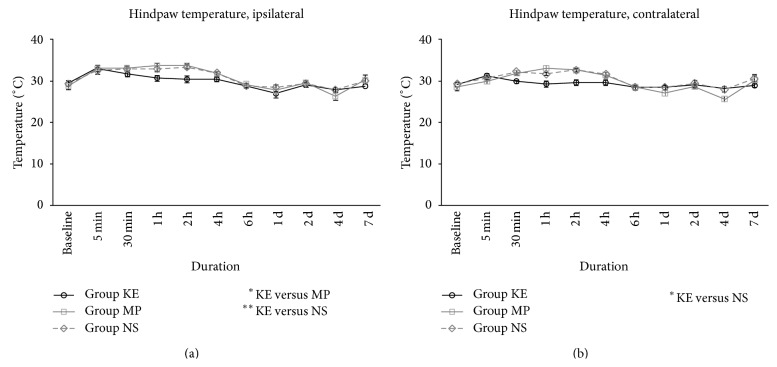
Hindpaw temperatures of KE, MP, and NS groups on the ipsilateral (a) and contralateral side (b) from baseline before ischaemia until the first 7 days after reperfusion. The temperature in group KE was lower in the ipsilateral side, compared with MP (*P* < 0.05) and NS (*P* < 0.01) groups. However, the difference was only obvious up to the 6th hour after reperfusion. Group KE also had decreased hindpaw temperature on the contralateral side during the same period, compared with group NS (*P* < 0.05); ^*^ *P* < 0.05 and ^**^ *P* < 0.01.

**Figure 2:**
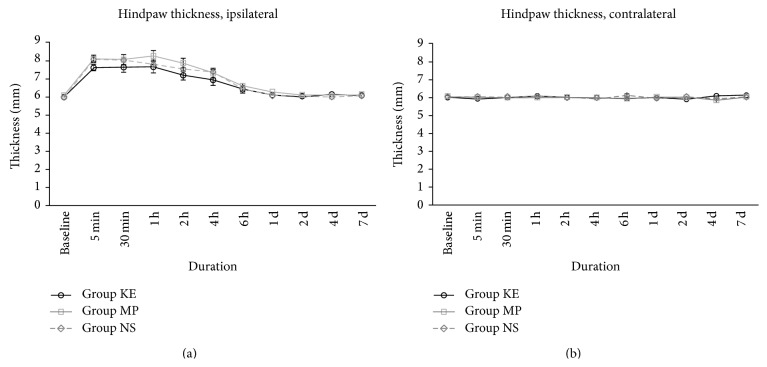
Hindpaw thickness of KE, MP, and NS groups on the ipsilateral (a) and contralateral side (b) from baseline before ischaemia until the first 7 days after reperfusion. There was no difference among all three treatment groups in the ipsilateral side during the 7-day study period.

Our thermography study showed that the temperature on the ipsilateral side hindpaws of group KE rats was lower than in the NS and MP group rats (*P* < 0.01 and *P* < 0.05, resp., Figure 1(a)). Ketamine also attenuated the rise in the hindpaw temperature on the contralateral side during the same period compared with group NS (*P* < 0.05, Figure 1(b)). Although there was a clear difference, it was only obvious up to the sixth hour after reperfusion. We also monitored the hindpaw thickness but no obvious difference was found between all three treatment groups in the ipsilateral and contralateral sides (Figures 2(a) and 2(b)).

### 3.3. Mechanical and Cold Allodynia

**Figure 3:**
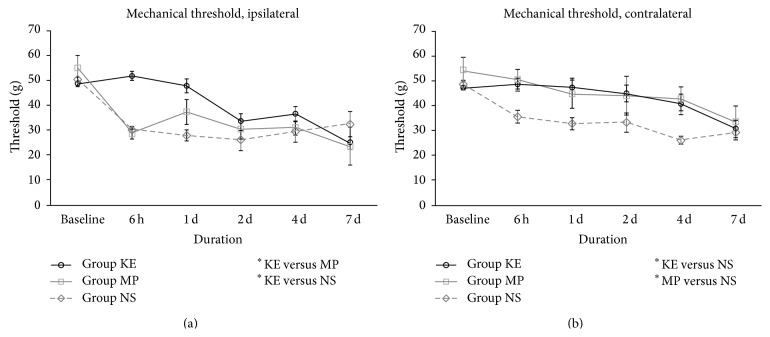
Mechanical allodynia of KE, MP, and NS groups on the ipsilateral (a) and contralateral side (b) from baseline before ischaemia until the first 7 days after reperfusion. On the ipsilateral side, the withdrawal threshold of group KE was significantly higher than that of NS (*P* < 0.01) and MP (*P* < 0.05). On the contralateral side, the withdrawal threshold was higher in both KE (*P* < 0.05) and MP (*P* < 0.05) groups than that in group NS; ^*^ *P* < 0.05; ^**^ *P* < 0.01.

**Figure 4:**
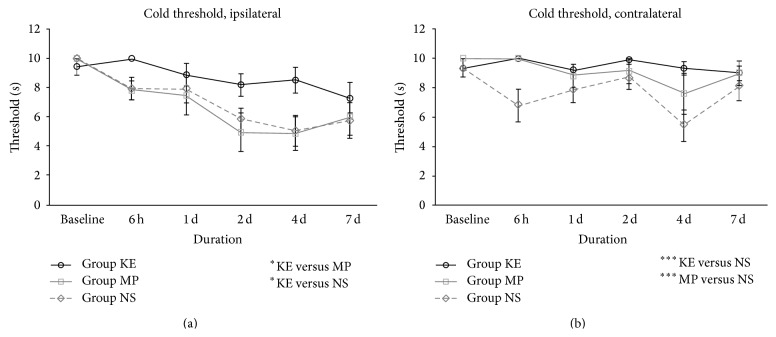
Cold allodynia of KE, MP, and NS groups on the ipsilateral (a) and contralateral side (b) from baseline before ischaemia until 7 days after reperfusion. On the ipsilateral side, the withdrawal threshold to acetone of group KE was significantly higher, compared with groups NS (*P* < 0.05) and MP (*P* < 0.05). On the contralateral side, the withdrawal threshold to acetone was significantly higher in KE (*P* < 0.001) and MP (*P* < 0.001) groups than that of NS; ^*^ *P* < 0.05; ^***^ *P* < 0.001.

Ketamine significantly alleviated both mechanical and cold allodynia in the ipsilateral hindpaw. On the ipsilateral side, the withdrawal threshold to the von Frey fibre in group KE was significantly higher than that in groups NS and MP (*P* < 0.01 and *P* < 0.05, resp., Figure 3(a)). On the contralateral sides, groups KE and MP also showed a higher withdrawal threshold to the von Frey fibre compared with group NS (all *P* < 0.05, Figure 3(b)). The withdrawal threshold to acetone in the ketamine treatment group was significantly higher on the ipsilateral side compared with group NS (*P* < 0.05) and group MP (all *P* < 0.05), as shown in Figure 4(a). For the contralateral sides, groups KE and MP also exhibited a significantly higher withdrawal threshold to acetone stimuli compared to group NS (all *P* < 0.001, Figure 4(b)).

### 3.4. Heat Threshold

**Figure 5:**
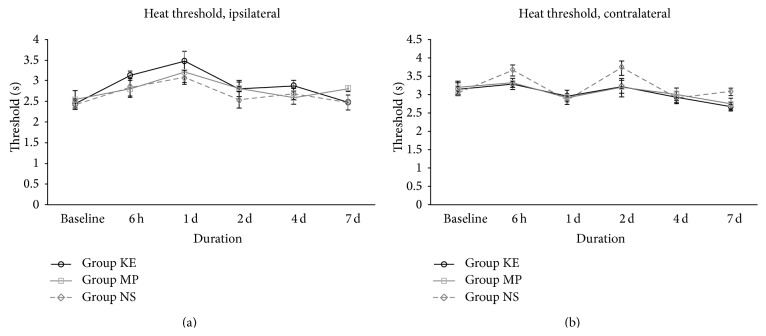
Heat thresholds of KE, MP, and NS groups on the ipsilateral (a) and contralateral side (b) from baseline before ischaemia to the first 7 days after reperfusion. There was no obvious difference of withdrawal threshold to fabricated radiant heat among all treatment groups in both ipsilateral and contralateral sides during the 7-day study period.

Fabricated radiant heat measurements produced no obvious difference in withdrawal threshold among all three treatment groups, in both the ipsilateral and contralateral sides (Figures 5(a) and 5(b)).

### 3.5. Serum TNF-*α* and IL-2

**Figure 6:**
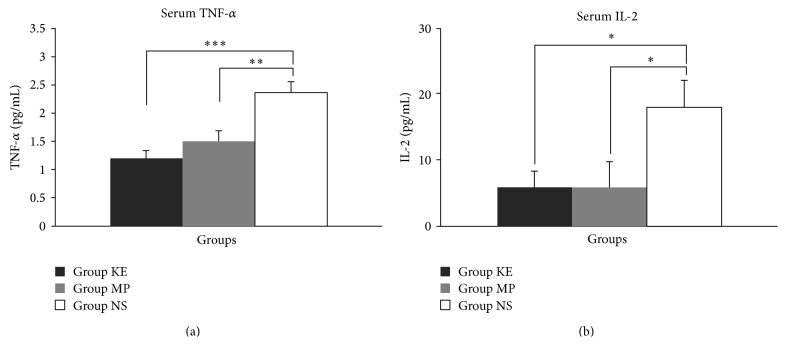
Serum TNF-*α* (a) and IL-2 (b) levels of KE, MP, and NS groups at the 48th hour after reperfusion. Compared with the NS group, serum TNF-*α* was significantly reduced in both KE (*P* < 0.001) and MP (*P* < 0.01) groups. Serum IL-2 was also significantly reduced in both KE and MP groups, compared with the NS group (all *P* < 0.05); ^*^ *P* < 0.05; ^**^ *P* < 0.01; ^***^ *P* < 0.001.

Groups KE and MP were able to markedly attenuate the increase in serum TNF-*α* levels (all *P* < 0.001, Figure 6(a)) and serum IL-2 (all *P* < 0.05, Figure 6(b)) forty-eight hours after reperfusion, compared with group NS.

## 4. Discussion

The present study successfully reproduced Coderre's CPIP model by inducing CRPS-I-like symptoms such as hyperaemia and mechanical and cold allodynia in both the ipsilateral and contralateral hindpaws of experimental SD rats. The appearance of bilateral symptoms has also been observed in other animal models of CRPS-I [18–21] and this is often a feature in patients with CRPS-I [22, 23]. The underlying mechanism of this contralateral effect may involve central sensitization caused by damage to muscle tissue [24]. It is thought that persistent inflammation after ischaemia-reperfusion injury may sensitize and activate afferent nociceptors in damaged tissue which may then lead to central sensitization contributing to the mechanical and cold allodynia observed in CPIP [12]. In this study, as medications were given immediately after induction of ischaemia, the treatment was essentially preventive of the acute phase of CPIP.

Ketamine is an NMDA receptor antagonist that also inhibits serotonin and dopamine reuptake, binds to *μ*-opioid receptors, and has effects on nerve growth factors and voltage-gated Na+ and K+ channels. It has been shown to produce analgesia by inhibiting normal and pathologic pain pathways, as NMDA receptor antagonism may attenuate central sensitization after tissue injury or inflammation [9, 25]. This may reduce secondary hyperalgesia and is reflected at the transcriptional level by a decrease in c-fos-oncogene induction. Ketamine has also been found to have anti-inflammatory effects [10], which may partially explain the analgesic effect in pain conditions with an inflammatory component, such as CRPS-I. It has been used for treatment of CRPS clinically [26–28] and it has been shown that a single infusion of intravenous ketamine improved pain relief in patients with critical limb ischaemia [29]. However, there has been no laboratory study conducted prior to our study to evaluate the potential mechanisms of its action on the development of CRPS and its analgesic action on CPRS. In the present study, both ketamine and methylprednisolone were used to explore their effectiveness on CPIP-induced CRPS. Methylprednisolone (a glucocorticoid with powerful anti-inflammatory effects) was used as a positive control to determine whether ketamine had additional therapeutic effects on this ischemia-reperfusion pain model beyond any anti-inflammatory mechanism. Systemic TNF-*α* and IL-2 were assessed 48 hours after reperfusion because our preliminary data (unpublished) demonstrated that the difference in both TNF-*α* and IL-2 was significant when compared with the sham group (without ischemia-reperfusion). TNF-*α* was also shown to peak at this time point.

Based on the results of a previous study, glucocorticoid treatment appears to be associated with attenuation of pain symptoms by inhibiting inflammation and reducing circulating inflammatory mediators [16]. Therefore, in this study, the mechanical and cold allodynia in the contralateral hindpaw were diminished by the administration of methylprednisolone. However, rats receiving methylprednisolone did not show a reduction in mechanical and cold allodynia in the ipsilateral (ischaemic) hindpaw when compared with the normal saline group. It seems that inflammation did contribute to postischaemic pain but that this was most likely to be only one of the mechanisms resulting in mechanical and cold allodynia in the ischaemic limb. Although it has been reported that glucocorticoids can inhibit symptoms of CRPS-II by preventing plasma extravasation [16, 29], oedema from local plasma extravasation was not significantly different between all three treatment groups in this study. Additional evidence indicates that inflammation is involved in the CRPS pathophysiologic mechanism. The pain produced by a low pH infusion into the normal, contralateral limb was similar to patients' who reported CRPS-I pain on the ipsilateral side [30]. Of the many pain-related inflammatory cytokines, only a limited number have been reported as being associated with neuropathic pain and CRPS. Willis proposed the involvement of inflammation in the CRPS pathophysiologic mechanism [31]. They demonstrated that the inflammatory mediators TNF-*α* and IL-2 were elevated in patients with CRPS-I [7]. In fact, TNF-*α* and IL-6 were also found in the local blister fluid of these patients [6].

In comparison with methylprednisolone, the therapeutic efficacy of ketamine has been shown to be superior in this study, and this finding most likely reflects its multimodal effects [14]. We found a decrease in serum proinflammatory cytokines TNF-*α* and IL-2, supporting an anti-inflammatory effect. Not only does ketamine relieve the major pain symptoms in most cases [32], but also it attenuates other inflammatory symptoms, including oedema and temperature [33]. Recently, a systematic review conducted by Dale and colleagues concluded that the intraoperative administration of ketamine could inhibit the early postoperative IL-6 inflammatory response [10]. However, pain relief was not one of the outcomes measured in the studies included in this review. Furthermore, in most studies ketamine was given at the induction of anaesthesia, but its role in modifying the ischemia-reperfusion response, including pain, has not been evaluated [10]. In this present study, we assessed whether the anti-inflammatory effects of ketamine could contribute to pain relief and preconditioning the early stage of CPIP after surgery to prevent further development of CRPS and confirmed the advantage of ketamine as an adjuvant analgesic.

Mechanismwise, Boettger and colleagues argued that dorsal root reflexes (DRR) might be linked to increased neuronal activity in the central terminal of primary afferent fibres and the aggravation of peripheral inflammation [34, 35]. Sensitization by inflammation leads to antidromic signalling in primary afferent neurons in DRR. Therefore, pain is being enhanced at one end, and neuropeptide release and inflammation are being aggravated at each cycle at the other [34, 35]. In addition, NMDA receptors were demonstrated to mediate responses of dorsal horn neurons to hindlimb ischemia in rat. NMDA receptors on the presynaptic membrane might act as autoreceptors for glutamate and enhance the incoming series of action potentials [36]. Furthermore, activation of postsynaptic NMDA receptors in the spinal cord by the increased release of glutamate from primary afferents can lead to central sensitization [37]. Thus, an NMDA antagonist such as ketamine blocks these receptors and might have significant effects in reducing the glutamate release from these terminals and further attenuating the inflammatory response [35].

In this study, ketamine administered soon after an ischaemia-reperfusion injury has been demonstrated to modify the postischaemic response with superior analgesic effects when compared to methylprednisolone and normal saline over the study period. Early hyperaemia followed by long lasting mechanical and cold allodynia resembles the two well-known phases of CRPS-I in humans [30, 38, 39]. We examined hyperaemia by measuring temperature in the hindpaws and there was an increase in temperature bilaterally in the first 4 hours after reperfusion which mimicked the sometimes brief hyperaemia seen in patients with CRPS-I. The fact that ketamine attenuated the increase in temperature in both hindpaws while methylprednisolone only affected the ipsilateral side suggests that ketamine has a more prominent effect on the hot oedematous stage in prevention of further progression of the disease. Apart from the anti-inflammatory effect, there are other mechanisms to explain these improved outcomes. Ketamine not only blocks the NMDA receptor [40], but also at a high dose blocks other receptors such as opioid and muscarinic cholinergic receptors [40]. It inhibits NMDA receptor-mediated central sensitization by blocking NMDA receptors, thereby reducing the mean opening time of the channel. It also decreases the frequency of channel opening by an allosteric mechanism [40]. Blockage of NMDA receptor-mediated sensitization results in attenuation of symptoms and signs, such as mechanical and cold allodynia. It is this action on the central nervous system that alleviates mechanical and cold allodynia, not only in the ipsilateral hindpaw but also in the contralateral hindpaw.

## 5. Conclusions

In conclusion, our study demonstrates that early treatment with ketamine can modify postischaemic responses resulting in less mechanical and cold allodynia and lower serum levels of proinflammatory cytokines including TNF-*α* and IL-2 in a CPIP model using SD rats. Although methylprednisolone can also reduce these cytokines, the analgesic efficacy was lower than that provided by ketamine. Early administration of ketamine can potentially be an effective approach in preventing further progression of pain conditions with an ischaemic and inflammatory pathogenesis, including CRPS. Clinical studies exploring the anti-inflammatory and analgesic effects of anaesthetic doses of ketamine after ischaemia-reperfusion injury are warranted.
